# Dinoflagellate Amphiesmal Dynamics: Cell Wall Deposition with Ecdysis and Cellular Growth

**DOI:** 10.3390/md21020070

**Published:** 2023-01-20

**Authors:** Alvin Chun Man Kwok, Wai Sun Chan, Joseph Tin Yum Wong

**Affiliations:** Division of Life Science, The Hong Kong University of Science and Technology, Clear Water Bay, Kowloon, Hong Kong, China

**Keywords:** cell wall, harmful algal blooms, cyst, dinoflagellates, amphiesma, cellulose, zooxanthellae

## Abstract

Dinoflagellates are a major aquatic protist group with amphiesma, multiple cortical membranous “cell wall” layers that contain large circum-cortical alveolar sacs (AVs). AVs undergo extensive remodeling during cell- and life-cycle transitions, including ecdysal cysts (ECs) and resting cysts that are important in some harmful algal bloom initiation–termination. AVs are large cortical vesicular compartments, within which are elaborate cellulosic thecal plates (CTPs), in thecate species, and the pellicular layer (PL). AV-CTPs provide cellular mechanical protection and are targets of vesicular transport that are replaced during EC-swarmer cell transition, or with increased deposition during the cellular growth cycle. AV-PL exhibits dynamical-replacement with vesicular trafficking that are orchestrated with amphiesmal chlortetracycline-labeled Ca^2+^ stores signaling, integrating cellular growth with different modes of cell division cycle/progression. We reviewed the dynamics of amphiesma during different cell division cycle modes and life cycle stages, and its multifaceted regulations, focusing on the regulatory and functional readouts, including the coral–zooxanthellae interactions.

## 1. Introduction

Dinoflagellates are the major microplankton group with cellulose deposition and are important primary–secondary producers in aquatic ecosystems, and the major causative agent of harmful algal blooms (HABs). Their autotrophic and mixotrophic nutritional modes allow them to exploit many niches and across trophic levels, with their mucilage and marine snow thecal cell wall contributing significantly to aquatic carbon flow.

Amphiesmal dynamics are orchestrated with dinoflagellate life cycle developments that are important determinants in algal bloom development and termination [1,2,3]. Coral endosymbiotic dinoflagellates form the primary production in coral reef ecosystems, with the amphiesma–host interaction dominating the symbiotic relationship. Dinoflagellate counterintuitive sensitivity to turbulence, as well as cellular growth, are wired to amphiesmal cortical calcium stores, activation of which will stimulate bioluminescence in bioluminescent species. The relative abundance and composition of dinoflagellates and diatoms have been regarded as an indicator of both short-term and long-term environmental change [4,5], and have ecosystem-wide consequences in biogeochemical cycling [6] and climate change [7]. We review on these regulatory and functional aspects, as well as putting forward amphiesma as a dynamic structure, and not a static cell wall as commonly depicted. Readers are referred to earlier reviews for amphiesmal ultrastructure [8,9,10,11,12].

## 2. Dinoflagellate Amphiesma and Ecdysis

**Figure 1:**
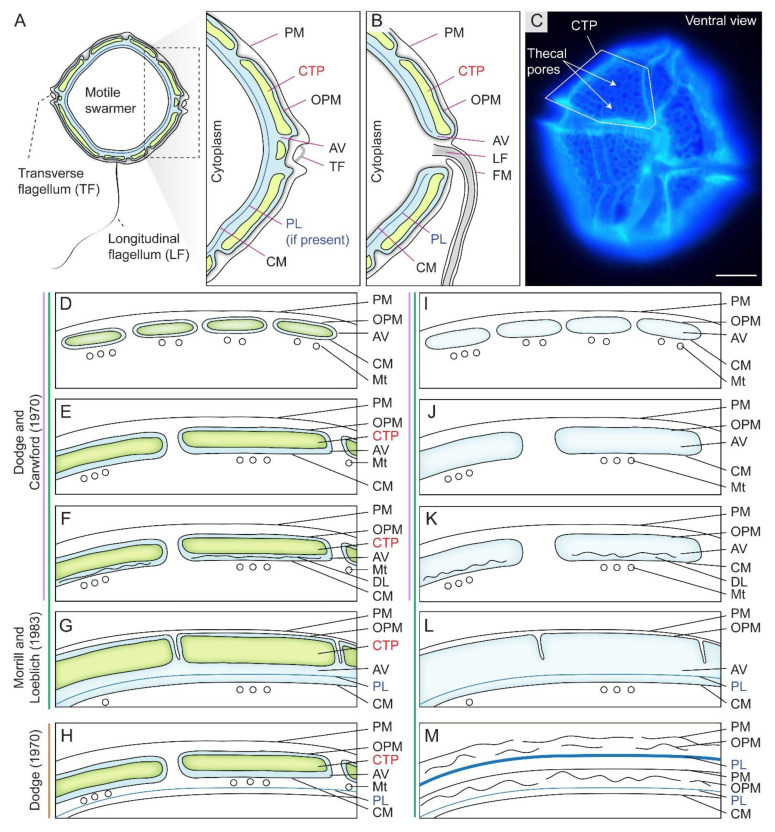
Diagrammatic representation of amphiesma arrangement in thecate and athecate dinoflagellates. (**A**) Schematic diagrams of the intracellular cell wall of a typical thecate dinoflagellate. Thecate dinoflagellate amphiesma consists of cellulosic thecal plates (CTPs), pellicular layer (PL or pellicle) and multiple membranous layers. PL is dynamic with vesicular–membrane attachment per different stage of development. (**B**) The outer plate membrane (OPM = outer plate membrane) and cytoplasmic membrane (CM = inner amphiesmal vesicle membrane) are joined at the flagellar region. (**C**) Fluorescence photomicrograph of Calcofluor white (CFW)-stained CTPs of a *Lingulodinium polyedrum* cell. In thecate species, CTPs could be encased either within (**D**) small contiguous (thin CTPs) amphiesmal vesicles (AVs), (**E**) larger contiguous AVs (thick CTPs), (**F**) contiguous AVs with discontinuous layer (DL), or in (**G**) a single-membrane-bound AV. The scheme proposed by Morrill and Loeblich (1983) differs from Dodge and Crawford (1970)’s proposal, by having an additional single-membrane-bound AV arrangement (**G**). The single-membrane bound AV in (**G**) is likely formed by fusing of adjacent AVs and discontinuous layers (as in **F**, and suggests the discontinuous layer may be the precedent of PL), resulting a continuous PL and CM. Athecate species share similar amphiesmal arrangement (**H**–**K**) as in thecate species, except without CTPs. Earlier study by Loeblich (1970) suggested that (**L**) PL is formed underneath AVs of thecate species, but Morrill and Loeblich (1983) later revised the model so that PL is formed within AV. (**M**) Multiple amphiesma in the (vegetative symbiotic) Symbiodiniaceae in hospite. Without completely discarding the old amphiesma, the new amphiesma is formed underneath the retained (but ruptured) old PM, OPM, and a (continuous) thickened PL. Examples for different amphiesmal arrangement: (**D**) *Woloszynskiu tylotu* [16] and motile cells of *Symbiodinium microadriaticum* [17]; (**E**)—*Heterocapsa pygmaea* [8], *Prorocentrum* spp. [18]; (**F**)—*Peridinium cinctum* [19], *Tripos muelleri* [20], *Lingulodinium polyedrum* [21]; (**G**)—*Crypthecodinium cohnii* [22], *Heterocapsa steinii,* [12], *Peridinium balticum* [23], *Scrippsiella trochoidea* [24], *Lingulodinium polyedrum* [25]; (**H**)—*Oxyrrhis marina* [26]; (**I**)—*Amphidinium carterae* [27], *Noctiluca scintillans* (sporocytes) [28]; (**J**)—*Karenia brevis* [29]; (**K**)—vegetative cells of *Pyrocystis* spp. [30], nonsymbiotic cells of *Symbiodinium microadriaticum* [17,31]; (**L**)—*Heterocapsa niei* [32]; (**M**)—Vegetative, symbiotic cells of *Symbiodinium microadriaticum* [17]. (**D**–**M**)—are adapted and redrawn with permission from [8]. Copyright © 1983, Elsevier (License number: 5471940090923). PM—plasma membrane (=outermost membrane); LF—longitudinal flagellum; TF—transverse flagellum; FM—flagellar membrane; Mt—microtubules. We adopt pellicular layer (=pellicle) to indicate the transient dynamic association between pellicular membrane and pellicular polysaccharides. Scale bar = 10 µm.

The dinoflagellate cell cortical membranous complex, termed amphiesma, is composed of two major interactive layers, the alveolar amphiesmal vesicles (AVs, also referred to as alveolar sacs or thecal vesicles) and the pellicular layer (PL), with different compositions during cell and life cycles [8,12] (Figure 1A,C). AVs compose flattened, circumcortical membrane-bound vesicle(s) underlying the plasma membrane [13,14], which are tightly entrained between the outer plate membrane (OPM, also referred to as outer amphiesmal vesicle membrane) and the cytoplasmic membrane (CM, also referred to as inner amphiesmal vesicle membrane) (Figure 1A,D,E). Individual cellulosic thecal plates (CTPs) are synthesized within AV(s) in thecate species (Figure 1D–H), whereas athecate (unarmored or naked) dinoflagellates have no CTPs (Figure 1I–L) and represent the simplest amphiesmal arrangement [8]. From this basic assembly, a vast diversity of forms and cell morphology, including round to cylindrical shapes, are formed [15]. The ultrastructure-based multilayered amphiesma was first described in Dodge and Crawford (1970) and Morrill and Loeblich (1983) [8,12], which have an outer membrane (plasma membrane, PM) in continuity with flagella (Figure 1B).

Dinoflagellates have complex life cycles with corresponding drastic amphiesmal remodeling, during which different layers take turns to become the surface-facing layer, and with different levels of associated polysaccharides or their precursors [33,34]. We adopt pellicular layer (=pellicle) without presumption of its membrane or/and polysaccharide composition, highlighting the transient dynamic association between pellicular membrane and pellicular polysaccharides. Either the motile flagellated swarmer cells or the non-motile coccoid cells are the major vegetative stage of free-living dinoflagellates. Swarmer cell AVs and CTPs are distal to the pellicular layer (PL) that is in contact with the innermost CM (Figure 1). The free-living heavily thecate dinoflagellate *Lingulodinium* (=*Gonyaulax*) *polyedrum* (=*polyedra*), a major model for bioluminescence studies [35,36] with a recently developed gene knockdown protocol for amphiesmal regeneration [37], was most comprehensively studied in relation to changes with life-cycle amphiesmal transitions [38,39] and will be taken as an example for the following discussion.

**Figure 2:**
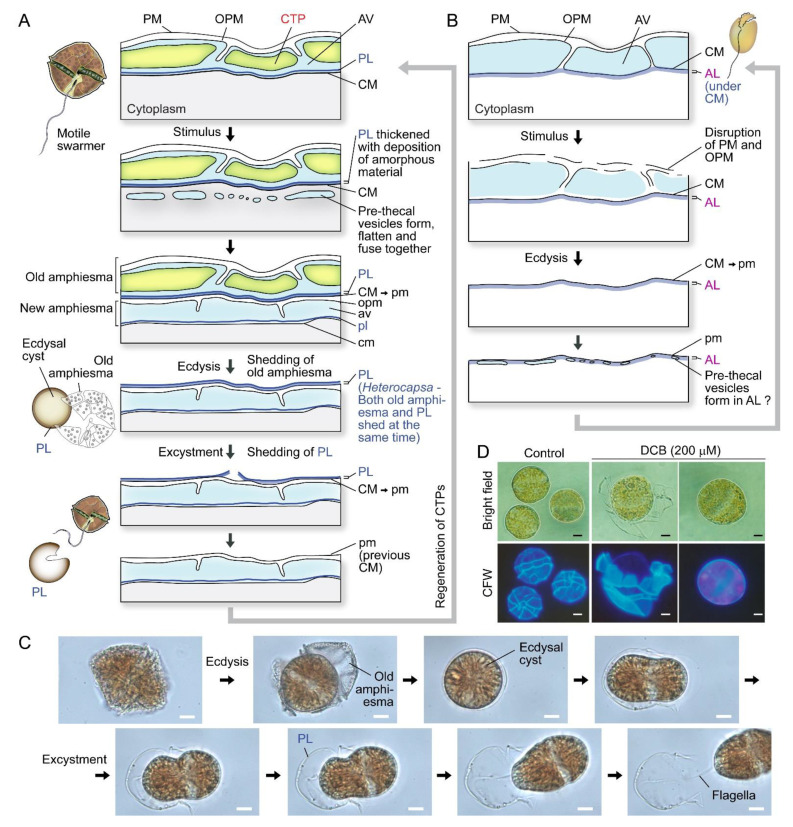
Amphiesma dynamics during ecdysis. (**A**) Diagrammatic representation of amphiesmal modifications during ecdysis. At early stage of ecdysis, pre-thecal vesicles formed and fused to form amphiesmal vesicle (AV) beneath the cytoplasmic membrane (CM). The old amphiesma, including the outermost plasma membrane (PM), outer plate membrane (OPM), and thecal plates (CTPs) would be lost, rendering the pellicular layer (PL) to become the new outermost layer [22,33,37,59,60]. The cell regained motility and regenerated CTPs soon after excystment. Whether the pellicular layer of the resulting cyst is already present in the motile cell beneath the theca likely depends on sampling timing and sample preparations. (**B**) In the non-pelliculate, athecate *Amphidium carterae*, an amorphous layer (AL) is formed beneath the CM in motile vegetative cells [49]. Unlike pelliculate species, CM and AL becomes the outermost layers, after shedding of the disrupted PM and OPM. New amphiesmal vesicles (AVs) appeared to form inside the AL. The uppercase letters refer to the old/parental amphiesma while the lowercase letters designate the new amphiesma components. (**C**) Photomicrographs of a *Lingulodinium polyedrum* cell going through ecdysis and excystment. PL is shed upon swarmer cell regeneration. See also Video S1. (**C**) and Video S2 were captured by Wing Tai Lam. Noted cell shape changes per excystment. (**D)** Photomicrographs of 2,6-dichlorobenzonitrile (DCB)-treated *Alexandrium catenella*. DCB induced shedding of thecal plates and formation of ecdysal cyst (round) cells. The outermost layer, at least in coccoidal stage, were PL with cell wall polysaccharide(s) and not the plasma membrane as depicted in some texts. The extracellular nature is thus equivalent with plant extracellular cell wall at this stage. Scale bar = 10 µm.

Ecdysal cysts (ECs), also referred to as temporary or pellicle cysts, are formed in response to stresses, in normal life cycles in many dinoflagellates (Figure 2A–C), including sexual temporary cysts [1,33,40]. Following ecdysis, PL becomes the new outermost layer [1,40,41,42], although the exact description could be different in relation to PL genesis, which had to involve vesicular transport and incorporation into the amphiesma. Subcytoplasmic “pellicular amorphous materials” and inner (new) amphiesmal vesicles were commonly formed after discarding their old cell coverings to form cysts [8,43,44]. In ecdysal cysts (or the transient coccoid cells), the old PL generally becomes the most distal layer but with a developing new PL underneath (or within) the developing AVs (Figure 2A). Athecate CM was reinforced with amorphous polysaccharide/cellulose, as cellulase treatment resulted in loss of cell shape [45], and those cellulose synthases were conserved amongst athecate and thecate dinoflagellates [46]. CM, with the underneath amorphous (polysaccharide) layer (AL), would become the outermost layer in athecate species following ecdysis (Figure 2B). Physical stresses (including centrifugation) induced ecdysis [37,47], indicating centrifugation-harvested “cell wall (amphiesma)” preparations will include a mixture of ecdysed and non-ecdysed cells, with likely transient expressed proteomes affected by membrane depolarization, rendering molecular and biochemical characterization interpretation difficult.

PL is a dynamic “working layer” during stress-induced ecdysis, with the inner amphiesmal vesicles membranes (=CM) fused with each other and form a continuous membrane (pellicle membrane) that remains attached to the underlying amorphous PL [48]. At different stages of development, PL varies in thickness and composition, including vesicular dense materials prior to deposition [33,44,48,49,50], and is not always present in spot harvested ecdysal cyst cells [8,51] (Figure 2A,C). This likely accounted for the discrepancies on the continuous or discontinuous nature of PL between previous ultrastructural studies [25,52,53]. The exact PL chemical nature is unknown, but there were cytochemical studies indicating nitrogen-rich glycans, chitin-like glycans, and non-cellulosic beta-glycans [54,55]. Discontinuous layers were described as a “thecal membrane” [20], a “discontinuous layer of dark-staining material” [56] or a “discontinuous pellicle precursor material” [8], which are interpreted as cells lacking the PL (e.g., *Tripos muelleri* [20,57], *Lingulodinium polyedrum* [21], *Gymnodinium simplex* [58], and ecdysing cells of *Heterocapsa niei* [8]).

Spatial-temporal amphiesma depositions will affect the residency of the outer membrane as glucoside bond formation requires an aqueous environment. There were no preformed polyssacharides (nor CTP) in the cytosol, indicating deposition involving in situ synthesis. The complete filling of the inner membrane front will inadvertently lead to displacement of aqueous domains, cessation of further distal deposition, and maintenance withdrawal of the outer membrane. This will lead to the membrane attrition (as depicted in Figure 1M), essentially rendering the attached pellicle as the outermost layer, despite apparent outer layers. Furthermore, this will initiate the inner thecal membrane to be the cell margin, as in other cells with extracellular walls, and harness the readiness for the continuum of AV-PL dynamics. Future investigations should relay how Ca^2+^ signaling is orchestrated with vesicular dynamics and polysaccharide deposition.

## 3. Cellulosic Thecal Plate Development Program

**Figure 3:**
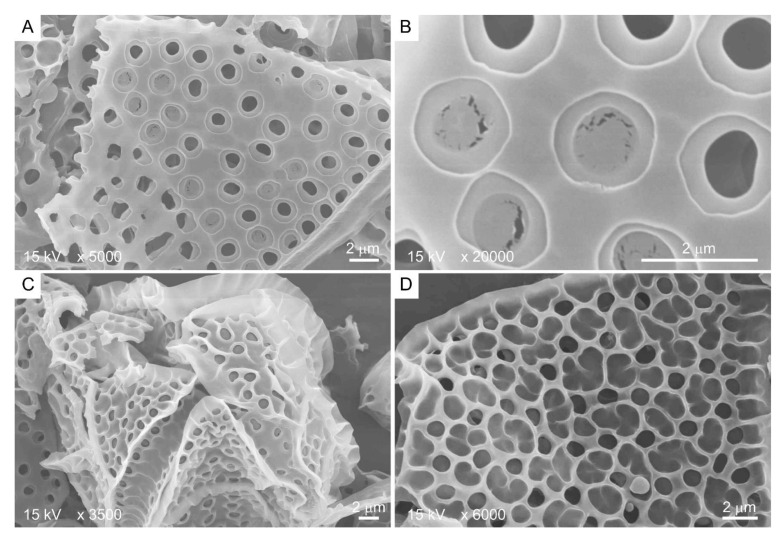
Scanning electron microscopy images showing the two different sides of *Lingulodinium polyedrum* thecal plates. (**A**,**B**) Cytoplasmic side contains thecal pits with flaps; (**C**,**D**) seaward side contains three-dimensional ridges as extension of thecal pit wall. Scale bars = 2 μm.

Kofoid thecal formula, the relative positioning of the thecal plate in relation to the cell surface, constitutes a major taxonomic character for thecate species [61], suggesting a non-random mode of deposition-biogenesis. Thecal plates very often have spectacular decorations, including thecal pits, sutures, and, in some species, with reticulate deposits [15]. CTPs could reach several hundreds of nanometers in thickness [62] and have two dissimilar sides: the distal (seaward) side, having thecal pores, thecal pits, ridges, and reticulates (Figure 3C,D), and, in some, a relatively smooth cytoplasmic side (Figure 3A,B).

**Figure 4:**
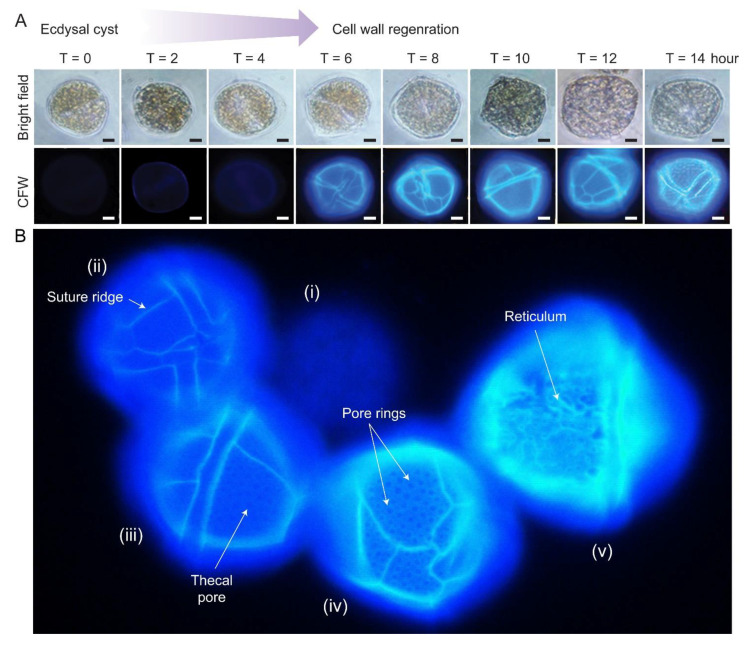
Cellulosic thecal plate regeneration after ecdysis. (**A**) Fluorescence photomicrographs of calcofluor white (CFW)-stained regenerating *L. polyedrum* ecdysal cysts, which were prepared as described [37]. Thecal plates were first observed at T = 6 after ecdysal cyst induction. Scale bar = 10 µm. (**B**) Fluorescence photomicrograph of CFW stained *L. polyedrum* cells with thecal plates regenerated at different stage of development. From left to right, (i) Ecdysal cyst cell with no thecal plate and completely lacked surface ornamentations; (ii) cell with weak CTP board; (iii) increasing CTP board and ridge deposition with the emergence of thecal pores/holes and (iv) the pore rings; (v) seaward side decoration (e.g., reticulum) was progressively added on the CTP board after ridge thickening.

There were apparent specific sequences of events during CTP development [63]. Fluorescence photomicrograph of CFW stained *L. polyedrum* cells revealed that the thecal plates regenerated at different stages of swarmer cell regeneration (Figure 4A,B). The ecdysal cyst had no thecal plate and completely lacked surface ornaments (i). Thecal plates were first regenerated with (ii) ridges along the sutures/plate border, then (iii) pores/holes (thecal pits) and (iv) pore rings, prior to the (v) formation of reticulum (significantly higher pore rings connected by ridges), including the three-dimensional thecal pit wall on the seaward side. Subsequent CTP board deposition, with turgor pressure bulging outward, would likely form the polygonal shape.

During vegetative growth, new cells commonly have less developed decorations than old cells and intercalary bands between plates [64]. No suture, and hence, no discernible plates, were observed on young thecae of *Alexandrium tamarense* and *Scrippsiella acuminata* [24,65]. Growth zones [66] suggest deposition expanded both laterally and vertically, with an overlapping region bulging outwards. Thecal pores are also less developed in young cells, suggesting deposition after thecal plate board, which is, in turn, followed by the formation of thecal ridges [42,44,66].

## 4. Amphiesma and Cellulose Deposition in Different Cell Division Modes

**Figure 5:**
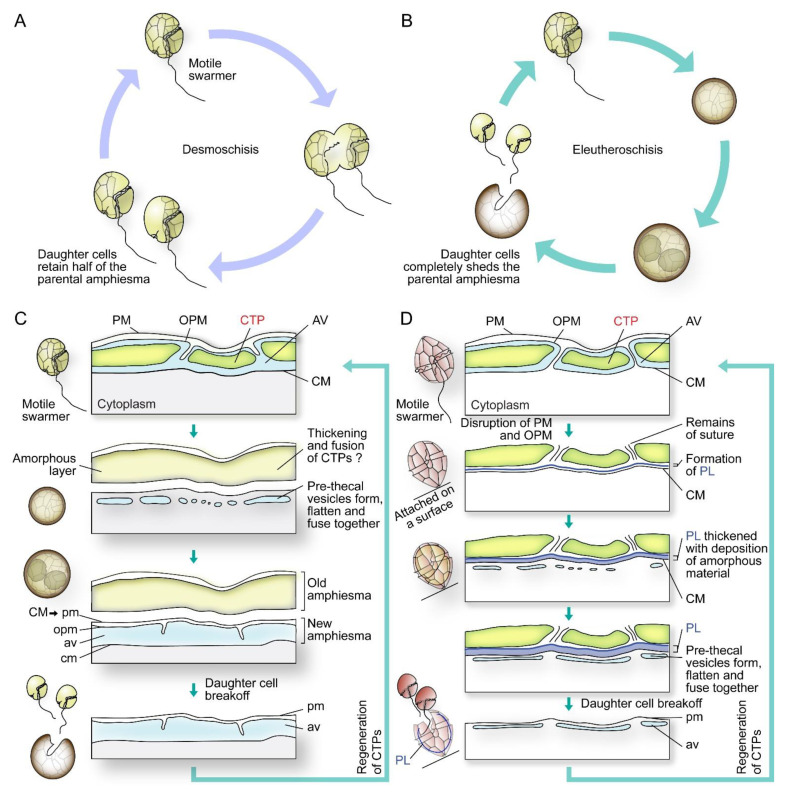
Diagrammatic representations of dinoflagellate cell division types and associated amphiesmal rearrangement. Dinoflagellate cell divisions involve (**A**) desmoschisis with half amphiesma regeneration or (**B**) eleutheroschisis with completely shedding of the parental amphiesma. (**C**) In one type of eleutheroschisis, for instance in *Crypthecodinium cohnii,* swarmers become deflagellated coccoid prior to cytokinesis within the mother cell, followed by daughter cells break-off [22]. Symbiodiniaceae in hospite undergone coccoidal eleutheroschisis without swarmer cell regeneration [89]. (**D**) In a second eleutheroschisis type, for instance in *Scrippsiella hexapraecingula*, the old thecal layer was attached after disruption of the plasma membrane (PM) and outer plate membrane (OPM) in ecdysal cyst that was attached on a substrate [44]. Daughter cells are formed inside the mother cell without apparent transformation into coccoid stage. It is apparent the different bioprocesses are modular in their sequel of events during amphiesmal dynamics.

In cell divisions, daughter cells generate the missing half through cytokinesis (desmoschisis) [59,67] or through eleutheroschisis, in which each daughter cell regenerates the amphiesma within the mother cell and then break off following cytokinesis (Figure 5A–D) [44,60,68]. The associated mobility switch with these life-cycle transitions commonly involved flagella regeneration (as in eleutheroschisis) or duplication (as in desmoschisis) [8,33,69].

**Figure 6:**
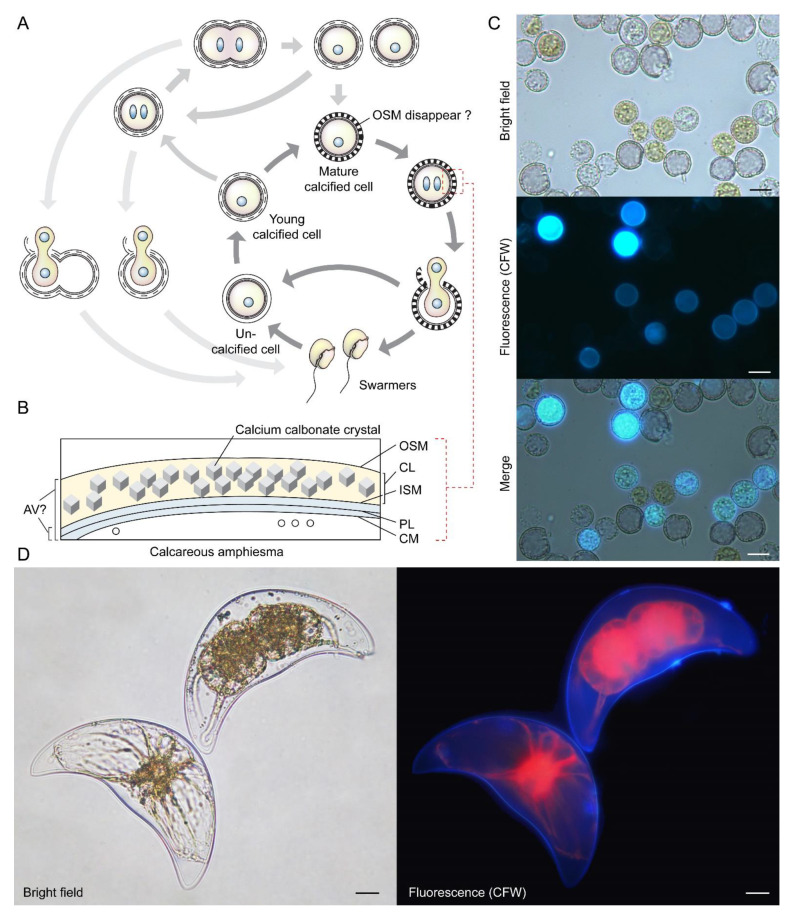
Coccoidal cell division in *Thoracosphaera heimii* and *Pyrocystis lunula*. (**A**) Diagrammatic illustration of the vegetative coccoidal apolar cell division cycle of *Thoracosphaera heimii*, which have surface carbonate deposition [71,72,90]. The calcified stage (coccoid), which can be covered by a thick perforated shell of calcium carbonate, is the dominant life cycle stage. Calcification appears to be uncoupled from cell cytokinesis; calcification could begin before the completion of cytokinesis, resulting in the appearance of twin-calcified shells. The outer shell membrane (OSM) is usually not visible in mature cells that have been fixed for electron microscopy, but these may be artifacts introduced by fixation. Redrawn with permission from [90]. Copyright © 1982, Elsevier (License number: 5472890419893). (**B**) *T. heimii* amphiesma is composed of the outer shell membrane (OSM), the calcified layer, inner shell membrane (ISM), pellicular layer (PL) and cytoplasmic membrane (CM) [71,91]. The location of AV is not clearly defined at present. (**C**) Fluorescence photomicrographs of calcofluor white (CFW)-stained *T. heimii* cells. CFW-positive suggested some stages contained polysaccharides layer. (**D**) Florescence photomicrograph of CFW-stained vegetative *Pyrocystis lunula* cell undergoing coccoidal division within the lunar-shape (vegetative) cyst wall. Lower left cell—before division; upper right cell—undergoing coccoidal division. Red = autofluorescence. Coccoidal division type does not strictly follow either desmoschisis nor eleutheroschisis, as it involved deflagellation as well as daughter cells regenerating half amphiesma, and we hereby term it Forchisis. Scale bar = 10 µm.

**Figure 7:**
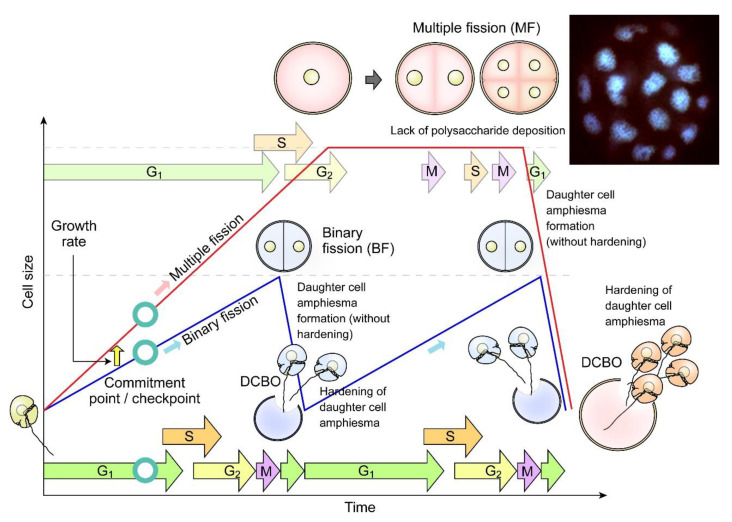
Diagrammatic representation of dinoflagellate multiple fission. Multiple fission (MF, pink cells) occurs when G_1_ cell size (cellular growth) has gone beyond double cell size, followed by n rounds of S phase-cytokinesis within mother amphiesma to generate 2^n^ cells without polysaccharide/thecal plate (CTP) deposition [81]. The daughter swarmer cells amphiesma CTPs will be hardened with apparent pre-deposition, shortly after daughter cell break-off (DCBO). Fluorescence photomicrograph (inset) of a DAPI-stained *Crypthecodinium cohnii* cell undergoing multiple fission. Sixteen nuclei are in focus, another sixteen are out of focus. The whole cell was about 100 µm across. Modified with permission from [81]. Copyright © 2005, John Wiley and Sons (License number: 5472221428122).

Coccoidal stages could also undergo divisions through apolar duplication–fission without going through swarmer cells, for instance in symbiotic zooxanthellae in hospite [70], in the carbonate dinoflagellate *Thoracosphaera heimii* [71,72], and in *Pyrocystis lunula* [73] (Figure 6A–D). These coccoidal fissional divisions [8,74,75,76] exhibited logistics similarity to the double PL layer arrangement in *Crypthecodinium cohnii*, after eleutheroschisis associated ecdysis (see later session). *C. cohnii* swarmers become a deflagellated coccoid prior to cytokinesis within the mother cell, followed by amphiesmal “hardening” after daughter cells break-off (DCBO) (Figure 5B,C). Deflagellation and reformation of flagella in these dinoflagellates thus appeared to operate with cellular duplication–fission. Desmoschisis, swarmer cell divisions without coccoidal transition and deflagellation, involves amphiesmal cellulosic deposition on one half of the cell, suggesting polarized deposition, in comparison to circumpolar deposition in eleutheroschisis. Polarized growth of CTPs was commonly facilitated by newly deposited apparently “unoccupied AVs” having higher vesicular incorporation propensity. In athecate cells (for example *Gymnodinium* species), the cleavage furrow separates the vegetative cells diagonally into left and right parts by desmoschisis [77] (Figure 5A). During multiple fission [78], the extended G_1_ growth phase was followed with S phase-cytokinesis without CTP deposition, which occurred shortly after daughter cell break-off (Figure 7). This suggested that cellulosic deposition was coordinated with cell cycle. As far as we know, there was no plasmodesmata reported for dinoflagellate cell division. Dinophysis “small and intermediate cells”, which are associated with nutritional downshift, are formed through depauperating fission that results in dimorphic daughter cells [79,80,81], demonstrating, again, the amphiesmal versatility in nutritional responsive polarized growth. The coccoidal division type does not strictly follow either desmoschisis nor eleutheroschisis, as it involved deflagellation as well as daughter cells regenerating half amphiesma, and we hereby term it Forchisis. Multiple fission, on the other hand, is an extended modification of eleutheroschisis, and we refer it to ex-eleutheroschisis.

The cellulose G_1_ level increased and halted before a slower G_2_ increase in the heterotrophic *C. cohnii* [60]. 2,6-dichlorobenzonitrile, an agent that purportedly inhibits the microtubular interactions with cellulose synthase complexes [82], dose-dependently disrupted CTP formation and cell cycle progression in dinoflagellate cells [60], despite there being no cortical microtubule organizing centers (MTOCs). Cellulases were required in cellulose deposition in both prokaryotic and eukaryotic cellulose deposition, for instance, the plantae endoglucanase KORRIGAN [83,84,85], and we demonstrated the cortically located endoglucanase dCel1p was required for cell cycle progression [45]. Cellobiose, a cellulase inhibitor, delayed dinoflagellate cell cycle, whereas recombinant endoglucanase dCel1p accelerated *C. cohnii* cells in coccoidal G_2_, indicating the extension of cellulosic layer through endo-cleavages of amorphous domains was involved during CTP growth [45]. dCel1 transcript level was upregulated in cell shape modulation, during responses to redox changes, in *P. lunula* [86]. Dinoflagellates with long amphiesmal extension (including *Ceratium* (now also *Neoceratium*) spp., *Ceratocorys horrida*) are well known to change morphology in relation to flow intensity [57,87] and digitation changes with light–dark cycle [88]. This is likely contributed to by vesicular transport being adapted in response to current flow, coupled with cortically associated cellulase dCel1p [45].

## 5. Polysaccharide Deposition during Amphiesma Dynamics

Cellulose, comprising parallel unbranched β-1, 4-linked glucan chains that form microfibrils, is the major reinforcing element of plant cell walls that provides mechanical strength [92]. Dinoflagellate cellulose synthase *dCesA1* knockdown led to cessation of ecdysal-swarmer regeneration [37], without flagella, suggesting cellulose synthesis dependency in the completion of amphiesma development.

*C. cohnii* amphiesma was stained positively for polysaccharides (CFW staining) but negatively for callose (aniline blue staining) [60]. The stringent chemical assay with the Updegraff protocol [93] demonstrated acid-resistant crystalline cellulose content being proportional to the CFW fluorescent signals (which also stained amorphous cellulose) [60], supporting the CTP nanomechanical hardness [62]. Earlier histochemical investigations using IKI/H_2_SO_4_ and zinc–chlor–iodide (Schultz solution), glucan assays with phenol sulfuric methods, and dissolution of isolated amphiesma preparations using basic solvents (e.g., 3%-NaOH, 100 °C for 5 h) [8,32,94] should be reinvestigated with more stringent assays, especially in relation to the co-staining of PLs.

**Figure 8:**
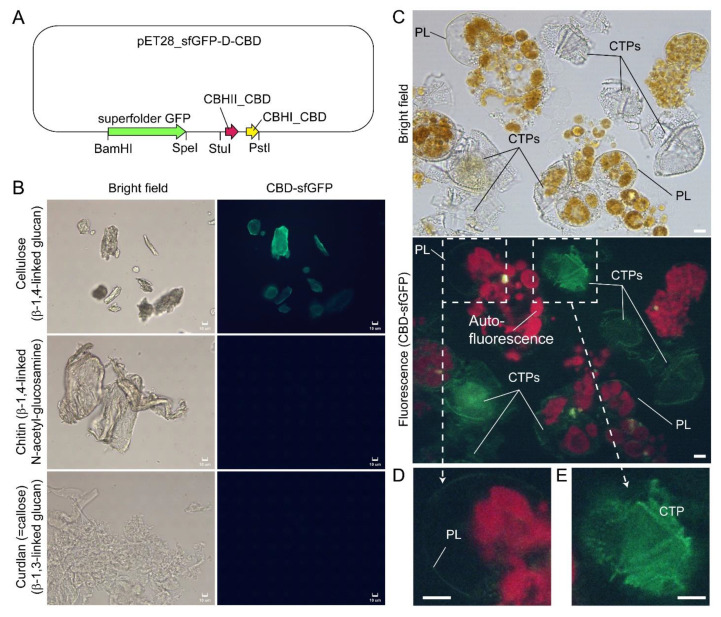
Compressed cell preparation of *Lingulodinium polyedrum* labeled with fluorescent recombinant cellulose-specific hybrid cellulose-binding domains. (**A**) Map of plasmid construct used for the generation of recombinant cellulose-specific carbohydrate-binding domain (CBD)-sfGFP fusion protein, which contained double CBDs as described in [97]. Neither single *T. reesei* CBHII CBD nor *T. reesei* CBHI CBD exhibited such specificity [96,97]. Double CBD protein was constructed by fusing the N-terminal (25–62 amino acids) of *Trichoderma reesei* CBHII CBD (AAG39980.1) to the C-terminal (478–513 amino acids) of *T. reesei* CBHI CBD (P62695.1) by a linker region of 24 amino acids (3 amino acid residues from natural CBHII linker followed by 21 amino acid residues from the natural CBHI linker). Fluorescence photomicrographs of CBD-sfGFP stained (**B**) microcrystalline cellulose, chitin, curdlan, and (**C**) *Lingulodinium polyedrum* cells (squashed gently). CTPs and pellicle (PL) were differentially stained green. Scale bar = 10 µm. (**D**,**E**) show higher-magnification views of the CBD-sfGFP-labelled (green) PL and CTP, respectively. These experiments also suggested previous single carbohydrate-binding domain non-specificity, of TEM CBD-gold labeling conducted in *Scrippsiella hexapraecingula* [42,44], could have labeled the PL. CTPs were strongly labeled, whereas PL was not labeled except along the broken rim and after extended exposure, which could be related to hydrophobic accumulation of the CBD domains that interact mainly by hydrophobicity.

In *Scrippsiella hexapraecingula* TEM preparations, the amphiesma was positively labeled with gold conjugated-CBHI (cellobiohydrolase I, source not mentioned, likely from *Trichoderma reesei*) and exhibited a cellulose type electron diffraction pattern [42,44]. Many cellulose-binding domain (CBDs), including bacterial CBDs (family II CEX from *Cellulomonas fimi*) and single CBHI CBD and single CBHII CBD from *Trichoderma reesei*, also bind chitin [95,96,97], and many “cellulase” preparations contained other hydrolase activities [98,99]. We further presented here CTP/PL binding with specific cellulose-binding domain (CBHI and CBDII CBDs, [96,97]) (Figure 8A–E). General polysaccharide dyes, including CFW, will not have this distinguishing staining. Ultrastructural studies concerning amphiesmal polysaccharides were interpreted from “electron dense materials” that could have been targeted to either the PL or the CTPs. TEM studies on *L. polyedrum* CTP biogenesis were reviewed in [10].

Given the highly dynamic nature of amphiesmal membranes, the strict interpretation of cytoplasmic membrane(s), should be taken with caution as to the transiency of all developing stages, as well as to whether thinly deposited membrane(s) commence with polysaccharide deposition. Key CTP biogenesis issues are the synthesis of non-round polygonal regularity with taxonomic precision being orchestrated with normal and apolar cellular growth.

Plant cell non-cellulose polysaccharides are pre-synthesized in Golgi prior to transport and exocytotic deposition [100]. Dinoflagellate amphiesma precursors were considered to originate from some small electron-dense cortical amphisomal vesicles, which moved to the periphery of the cell, flattened, and fused together [33,57,59]. CFW readily stained CTPs/amphiesma of *Alexandrium* (*Gonyaulax*) *tamarensis* but did not label internal compartments [101]. Similarly, the lack of CFW staining in any intracellular compartments in *C. cohnii* and *L. polyedrum*, except in AV and PL [37,60], indicated there were no or undetectable matrix polysaccharides in the vesicular transport pathway.

Plant membrane-targeted cellulose synthases complexes (CSCs) catalyze glucose polymerization from the substrate UDP-glucose into cellulose polymer. The rosette CSC archetypes originated late in the chlorophyte lineage, whereas the linear archetypes remained in the non-green lineages [102,103], as was reported in dinoflagellate *Scrippsiella hexapraecingula* (although single CBD domains were deployed) [42,44]. The prominent CTPs and availability of the cyst-generation method [43,47,52], in combination with CFW-assisted flow cytometry of cellulose content in dinoflagellate cells [60], facilitated biochemical investigations of cellulose synthesis (CS) dynamics during cyst-swarmer cells transition (T_c-s_) in *L. polyedrum*. Dinoflagellate *LpCesA1* transcript was upregulated 14-fold in the early stages of ecdysal cyst regeneration, with CTPs fully regenerated between 12 and 16 h [37]. *LpCesA1* antisense knockdown in *L. polyedrum* led to abnormal thecal plate deposition and postponement of the swarmer cell regeneration [37].

Amongst the alveolates, only dinoflagellates acquired cellulose synthesis [104]. Phylogenetic analysis led to the proposal of dinoflagellate cellulose synthase having a single origin [37]. It was highly unlikely that a CSL (cellulose synthase-like) glucan synthase would have co-evolved in the dinoflagellate lineage, as it required strict coordination of vesicular transport, and that most CSL genes were associated with land plants. Bacterial cellulose synthesis involves multiple non-cellulose synthase subunit complexes and supporting periplasmic proteins (e.g., BcsB, BcsF, and BcsZ) [105], but we could not find potential homologues from dinoflagellate transcriptomes, nor from published genomes (data not shown).

## 6. Amphiesma Dynamics and Vesicular Transport

Polysaccharide deposition requires vesicular transport of either in-vesicle pre-synthesis or vesicular transported cellulose synthase (CesA) that mediated *on*-plasma-membrane biogenesis [106]. Ultrastructural studies suggested polyvesicular bodies (PVBs, large endosomes) commonly located close to or attached to the alveolar sacs [44,107] with fusion of these vesicles with CM constituting amphiesmal biogenesis [108].

**Figure 9:**
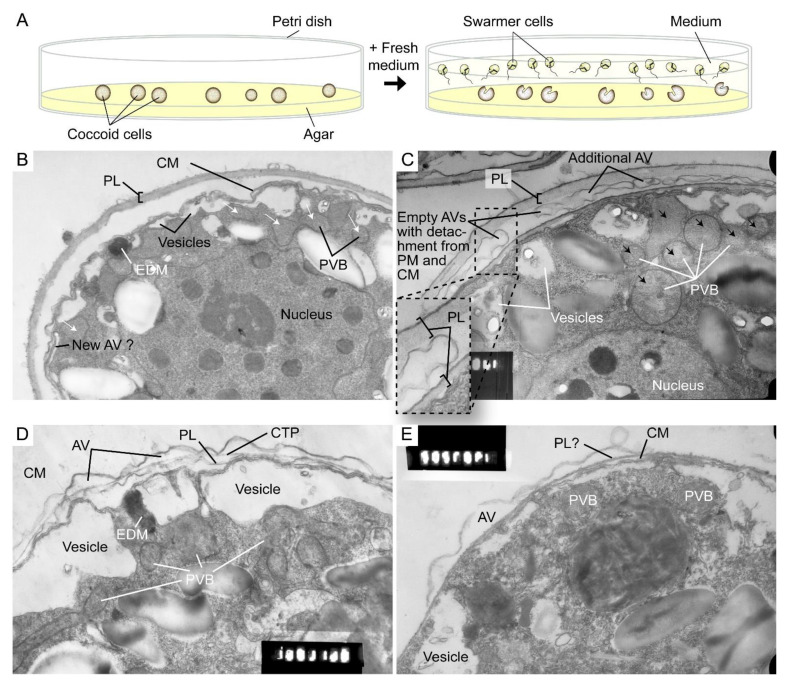
Amphiesmal rearrangements in coccoid cells after induced membrane fusion. (**A**) Schematic diagram showing the swarmer (daughter) and (mother) coccoid *Crypthecodinium cohnii* cells obtained by the coccoid-swarmer-release and filtration method [118]. For polyethylene glycol (PEG) treatment, cells were resuspended in 20% (*w*/*v*) PEG, before being spread on MLH agar plates. Transmission electron photomicrographs of the amphiesma in (**B**,**C**) coccoid (on agar plate) and (**D**,**E**) swarmer *C. cohnii* cells. Amphiesma of (**B**) control coccoid cell; (**C**) A PEG-treated coccoid cell; (**D**) A control swarmer cell and (**E**) swarmer cell released on PEG-treated plate. PEG treatment, which increased membrane fusion events [109], led to increased appearances of larger PVBs (polyvesicular bodies, large endosomes, black arrows in (**C**)) comparing to the smaller vesicles (white arrows in (**B**)) in control coccoid cells. It also drove thicker pellicular layer (PL) and amphiesmal rearrangement in the PEG-treated coccoid cell (**C**). The PL in PEG-treated mother cell exhibited a variation from apparently one layer with polysaccharide deposition (left) to two separate membranous layers with inter vesicular bodies (unfused, right); there were also lesser stained attached vesicular bodies outside the cell. TEM sections were in the same series that were published [109] and examined with a JEOL 100CX transmission electron microscope. EDM—electron dense materials. Magnification = 19,000×.

The highly dynamic amphiesma with vesicular transport was demonstrated in the polyethylene glycol (PEG)-treatment of on-agar coccoidal cells [109] (Figure 9A–D) with which membranous layers appeared displaced when compared to control cells. Coerced cortical membrane fusion (Figure 9B) was observed with accelerated vesicular transport resulting in dramatic amphiesmal rearrangements [109], demonstrating the non-permanent amphiesmal nature with sustained vesicular transport dynamics.

The coerced increase in fusion events [109] drove the disappearance of small vesicles and the accumulation of dense material in daughter swarmer cells, demonstrating the continuum of amphiesmal dynamics with the vesicular system in mother–daughter amphiesmal transition (Figure 9C,D). The small vesicles in the control cells were shifted to large peri-vesicles (~4.7 times increase in volume, as measured by ImagJ) in PEG-treated cells (Figure 9A,B). The coerced fusion of the outer layers (Figure 9D) exhibited similarity to the zooxanthellae cell wall in hospite [110]. PEG-treated mother cells exhibited a PL thickness variation within the same cell, from apparently one layer (left) to two separate membranous layers with inter vesicular bodies (unfused, right) (Figure 9B), indicating PL deposition involving two membranes. There were also lesser electron dense attached vesicular bodies outside the cell, substantiating the effect of extracellular PEG in driving vesicular transport, and seconding the potential role of secretion (e.g., muco-polysaccharides) in driving vesicular transport through the decanting of cortical vesicular membranes.

**Figure 10:**
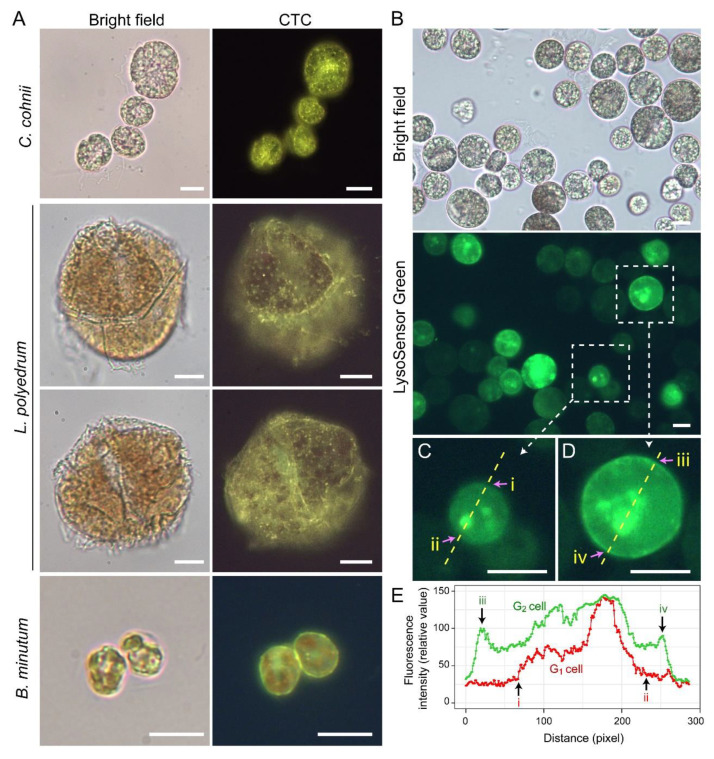
Amphiesma calcium stores and acidic compartments. (**A**) Fluorescence photomicrographs of chlorotetracycline (CTC)-stained *Crypthecodinium cohnii, Lingulodinium polyedrum* and *Breviolum minutum* cells. Cells were briefly fixed with 1% (*w*/*v*) glutaraldehyde in seawater (5 min, 22 °C) before CTC (excitation: 380 nm, emission: 520 nm [119]) staining with brief fixation protocol [120]. Over-fixation will lead to diminishing of subcellular CTC staining, suggesting the Ca^2+^ stores were associated with active vesicular transport. CTC-positive stores were observed on the surface and distributed over the cortical layer of the cell. In addition to the tiny-dots staining pattern, CTC also stained a continuous layer in the amphiesma (yellowish-green color). CTC localization in amphiesma could be affected by inter-membrane zeta-potential and may not specially require specific Ca^2+^ binding proteins. The red fluorescence is chlorophyll autofluorescence from chloroplasts. (**B**) LysoSensor Green DND-189 (excitation: 443 nm, emission: 505 nm, 2 µM, ThermoFisher) staining yielded fewer, but larger, dots/patchy labeling in *C. cohnii*. Both cell surface and subcellular compartments were stained, with apparent increased cortical labeling in larger G_2_ cells. (**C**,**D**) show higher-magnification views of a smaller G_1_ and larger G_2_ LysoSensor-stained cells, respectively. The boundaries of the G_1_ cell shown in (**C**) and G_2_ cell shown in (**D**) were marked by (i, ii) and (iii, iv), respectively. (**E**) Quantification of fluorescent level along transects in (**C**,**D**). Smaller G_1_ cells appeared to have less cortical labeling when compared to the larger G_2_ cells. In either case, there were associations of inner acid compartment with the nucleus. Scale bar = 10 µm.

Lysosensor probes, which are highly pH-sensitive, strongly labeled dinoflagellate cortices coinciding with the amphiesma (Figure 10B). Smaller G_1_ cells appeared to have less cortical labeling when compared to the larger G_2_ cells (Figure 10C–E) [111]. pH gradients are an important regulatory axis in the vesicular transport/secretary pathway, affecting all aspects including cargo sorting and protein processing [112,113,114,115], indicating the amphiesma’s acidic pH could act as a cellular growth-deposition driver. The association of _CTC_[Ca2+]^S^ (next section) further indicated amphiesma as a major homeostatic hub, having biochemical–biomechanical interactomes between the extracellular and intracellular environments. We do not adopt acidocalcisomes to emphasize the compartments likely different from vacuolar regulation, as lysotracker and CTC staining may not fully overlap (Figure 10A,B). The balancing of growth, with vesicular transport, with ecdysis-attrition through secretion and oxidative potentials, will be most evident in cells with apolar–circumpolar vesicular deposition.

Microtubules are believed to play a role in thecal development [43,60], despite there are no cortical MTOCs and the cell exhibiting no apparent dynamics; they likely form a network with alveolin homologues as reported in other Alveolates [14,116,117]. Amphiesma were shed in DCB-treated dinoflagellate cells (Figure 2D) [60], an inhibitor of cellulose deposition through severing microtubular contact [82]. Actin cytoskeleton was involved in plant cellulose deposition, but cytochalasin D, an actin polymerization inhibitor, exhibited no effect in the *C. cohnii* cell growth progress (Chongping Li, unpublished data). The eleutheroschisis lack of unidirectional cytosol expansion, as required in desmoschisis, could thus directly reflect growth–vesicular transport through the whole genome-growth cycle, as there is no nuclear envelope breakdown. This was demonstrated with extracellular PEG coercing amphiesmal cortical layer emptying, rather than a selective increase in AV board thickness, suggesting the dynamic amphiesmal with exocytotic vesicular movement directly drives intracellular movement of vesicles (PVBs), the depletion of which led to empty AVs with detachment from the plasma membrane and the cytoplasmic membrane (Figure 9).

## 7. Calcium Signaling in Ecdysis, Cellular Growth and Bioluminescence

Cellular growth rate-dependent cADPR-Ca^2+^ signaling pathways, including dose-dependent _CTC_[Ca2+]^S^ depletion, were demonstrated to orchestrate relative dinoflagellate cell growth, whereas cADPR-Ca^2^-store depletion mediated cortical mechanical sensitivity in dinoflagellates [78,120]. _CTC_[Ca2+]^S^ mobilization exhibited pharmacological characteristics of the ciliate subplasmalemmal-like Ca^2+^ stores, a special cortical endoplasmic reticulum [121,122] that exhibits Ca^2+^ level restraint overflow from external rise [120,123]. IP_3_-Ca^2+^ signaling inhibition led to ecdysis in dinoflagellate cells [124,125,126], whereas Dantrolene (antagonist of both Ryanodine (RyR) and IP_3_ receptors) efficiently blocked shaking (caffeine)-induced Ca^2+^ transient. Caffeine (cADPR receptor agonist) dose-dependently accelerated Ca^2+^ transient and plasma membrane deposition, resulting in an increase in relative cell sizes [120]. Whereas cADPR activates Ca^2+^- SERCA to Ca^2+^ influx from cytosol, cADPR and inositol 1,4,5-trisphosphate (IP_3_) commonly operate with sensitizing luminal Ca^2+^ gating of RyRs/IP_3_R to store overload-induced Ca^2+^ release (SOICR) [127,128]. Inhibition of either one will modulate the other [129,130,131].

**Figure 11:**
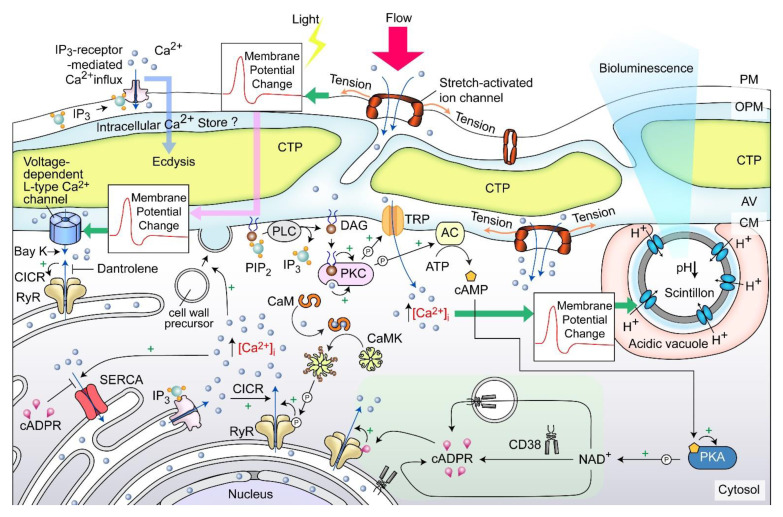
Amphiesma and calcium signaling. A diagrammatic representation illustrating our observations and hypothetical amphiesmal Ca^2+^ signaling pathway. The positions of the scintillon and the CTC-positive Ca^2+^ stores are arbitrary. RyR—Ryanodine receptor; PIP_2_—phosphatidylinositol 4,5-bisphosphate; IP_3_—nositol-1,4,5-trisphosphate; IP_3_R—IP_3_ receptor; DAG—diacylglycerol; SERCA—sarco/endoplasmic reticulum Ca^2+^-ATPase; CICR—calcium-induced calcium release; CaM—calmodulin; CaMK—Ca^2+^/calmodulin-dependent protein kinase; cADPR—cyclic ADP-ribose; CD38—ADP-ribosyl cyclase/cyclic ADP-ribose hydrolase; TRP—Transient receptor potential; cAMP—cyclic AMP; PKC—phospholipase C; AC—PKA—phospholipase A, ⓟ—phosphate/phosphorylation.

A dinoflagellate proton ATPase kHV1, which operated with negative Nernst potential [132,133], was proposed to function in the activation of the amphiesma associated scintillons-bioluminescence (Figure 11). Mechanically induced calcium release from intracellular Ca^2+^ store acts through the L-type Ca^2+^ channel (Figure 11), indicating the circuitry of vesicular H^+^-ATPase and L-type Ca^2+^ channels, as was shaking induced bioluminescence and mechanically induced ecdysis [107,134,135]. PLC inhibitor U73 122 blocked mechanically induced bioluminescence and indoleamine-induced IP_3_ production in dinoflagellate cells [126,136], indicating also the IP_3_ signaling involvement.

Mechanical shaking or the presence of fluidic mechanical forces inhibited cell proliferation of many dinoflagellates [137,138,139]. Each CTP within the surface orthogonal network of the amphiesma, with underlain cortical microtubules likely part of the mechanical sensitive system (as discussed earlier) responsible for sensing flow direction [140], and sustained stimulation could lead to depolarization, and, in turn, ecdysis or bioluminescence. This has similarity to the ciliate cortical AV-trichocyst system that is also based on AV Ca^2+^ signaling, in regulating cilia beating, including reverse swimming direction [141,142]. The intertwining between ecdysis, cellular growth, and scintillons indicates a potential bioluminescence role in dissipating oxidative stresses, as was proposed in the “oxygen defense” hypothesis [143].

## 8. Amphiesma in Hospite

The endosymbiotic relationships between Symbiodiniaceae members and corals form the photosynthetic basis of the coral reef ecosystem. Coral zooxanthellae are generally in coccoid forms in hospite; thinly thecated mastigotes (free swarmer cells) were reported in laboratory culture and for some species in situ [17,110]. Amphiesma from free-living *Symbiodinium microadriacticum* coccoid cells were demonstrated to contain polysaccharides by CFW staining following cellulase treatment, and a thin outer layer was interpreted as dinosporin [144]. Nonetheless, there were reports of pectinases-resistance in the Symbiodiniaceae cell wall [145]. Zooxanthellae plastids are located circum-cortical [110] as a single tubular organelle attached to amphiesma; amphiesmal dynamics will need to be negotiated with the host acidic symbiosome pH [146,147] that will affect chloroplast vesicular transport, as well as other vesicular components. These are important arrangements, as photosynthesis will likely elaborate high redox potential across the cortice–inner cytosol and will add on to the low pH of the symbiosome membrane as a driver for vesicular transport. Bearing in mind the cortical location of major dinoflagellate acidic compartments (Figure 10B), the dynamics of pH between the symbiosome membrane and amphiesma will be strategic in understanding the symbiontic relationship. As Lysosensor and Lysotracker dyes accumulate on membranes (both host and symbiont), comparisons should only be made between membranes or vesicular compartments, and not “zeroed” with empty cytosol, as was in [146,147], in the estimation of symbiosome pH.

In free-living *Breviolum minutum* cells, the (CTC)-positive Ca^2+^ stores labeled the amphiesma strongly, but higher than the internal compartments (unlike the other dinoflagellates), cortical to the chloroplast autofluorescence (Figure 10A), indicating their regulatory roles. Proposed host regulation of symbiosome pH would likely affect _cyt_[Ca2+] (cytosolic calcium)-_CTC_[Ca2+]^S^ (chlortetracycline (CTC)-positive Ca^2+^ stores) coupling and the corresponding vesicular transport, and orchestrate symbiont cellular deposition (or coerced passage) to the host cell. Intracellular-cortical trafficking is fundamental for transport of membrane proteins to the symbiosome membrane, as well as for lipid bodies maturation [148,149,150,151]. Low pH and redox potential will not only drive vesicular transport, but also affect the activation and sensitivity of _CTC_[Ca2+]^S^ [152]. Unwarranted acidic amphiesma will likely be pivotal in ecdysal regulation, the dysfunction of which likely causes ecdysis in hospite. Several lines of evidence indicated some symbiotic dinoflagellates likely undertake ecdysis-like events, including multiple layers of electron dense layers surrounding the symbiosome [17,89], symbiosomes with over nine membrane layers, multiple host non-symbiotic vacuole [153], and excess membrane vacuolation [154]. There were also several reports that Symbiodiniaceae in hospite had undergone coccoidal eleutheroschisis with discarded amphiesmal materials (forming multiple amphiesma (Figure 1M)) [110], but whether this was attributed to stresses was not addressed [89]. Steep redox/pH gradients will register higher sensitivity to unwarranted pH gradients, heat, or UVr (UV radiation) membrane damage, which could have led to cell size dysregulation that was identified as an early sign multivariate analysis [155,156] of coral bleaching.

## 9. Development of Resting Cysts Amphiesma

“Resting cysts” generally refer to the coccoidal product of sexual reproduction, of which there are also sexual temporary cysts [1,157], and cyst-to-swarmer transition plays prominent roles in dinoflagellate bloom dynamics. In most dinoflagellate lineages, coccoid cells accommodate swarmer zygote (hypnozygote) differentiation to resting cysts [158].

Resting cyst development generally takes longer than ecdysal cysts, resulting in thecal layer removal within the transient mucilage wall. *L. polyedrum* resting cyst development was described in detail in [39]. The basic modular units of AV-PL dynamics were adapted in resting cyst development, encompassing deflagellation, inflation of the outer plate membrane, endophragm formation, thecal plate dissociation, and globule-spine formation within the expanded central cell mass, followed by rupture of the transient mucilage and release of the resting cyst. The cyst mucilage wall, in some species, has surface reticulates [61]. It is generally regarded that spines developed from globules, or mucofibrous bulges in some species, which in turn developed from underlying thecal vesicles. Some thecate species produce resting cysts with paraplates, such as the *Gonyaulax spinifera* species complex and *Alexandrium pseudogonyaulax* [159,160], whereas in some athecate species, the thecal vesicle pattern could be replicated to the cyst wall [77] which commonly does not have spines. The cyst mucilage, which could be attached to young cysts, contained sulphur and silica [161]. In addition to dinosporin, the young resting cyst wall was purported to contain, based on histochemical interpretations, cellulose (internal), pectic compounds (pectin, exterior), and callose (which appeared later and was possibly associated with germination) [32,162].

The long resting cyst developmental periods were often classified into “young” and “old” periods, differing in aspects of surface deposition [77], which very often are highly decorated with spines and containing the chemically resistant dinosporin. Dinosporin was named attributed to the chemical resistance of the resting cyst cell wall, as in the sporopollenin of plant pollens, and may have more than one chemical that contributed to controversy [163,164]. Bearing in mind resting cysts amphiesma are likely composed of more than one layer, with some ultrastructural investigations demonstrating inner and outer cyst walls [165], we cannot exclude the occurrence of more than one major compound. Transition to resting cyst is a key ecological event in bloom termination that seeds the following annual cycle in many HAB species [1]. Sexual reproduction also involved diploid planozygotes, which undergo vegetative proliferation in some species [166] (Figure 18 of Gao et al., 1989) prior to transforming into either sexual ecdysal cysts or resting cysts, and with circadian dependency as reported for asexual ecdysis [38,167]. Most earlier literature can be found, described in detail, in [10].

## 10. Mucus Production

**Figure 12:**
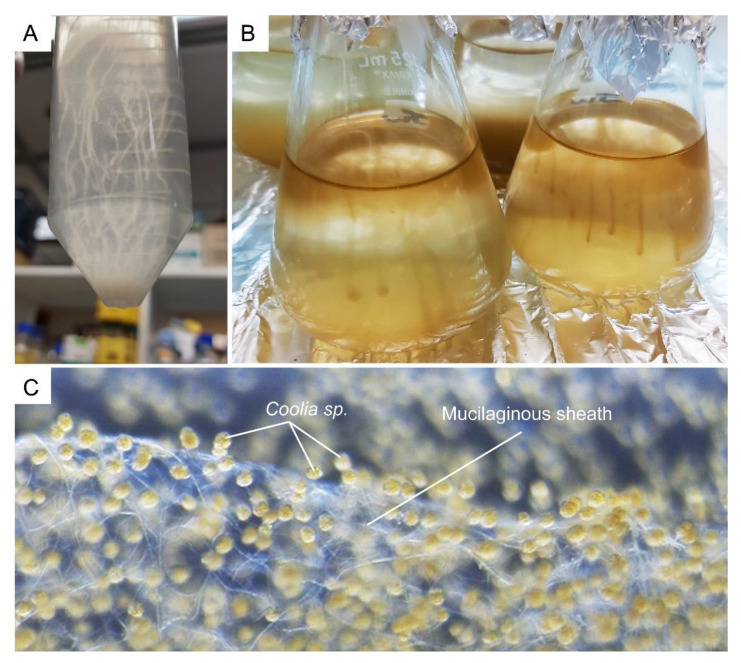
Dinoflagellates muco-polysaccharide. (**A**) *Crypthecodinium cohnii* and (**B**) *Amphidinium carterae* cells form strand-like cluster in suspension culture. (**C**) Complex network of tiny mucilaginous filament (sheath) was produced by the benthic *Coolia* sp. (family Ostreopsidaceae), which enables them to colonize benthic substrates. See also Video S2. The *Coolia* sp. was cultured in 24 well plate, with f/2 medium at 22 °C for a month. The amorphous polysaccharidic component of mucilage was proposed to derive from pusule fibrous material and mucocysts [174].

Dinoflagellates produce a wide spectrum of toxins, which require passage through the amphiesma, assuming an extracellular role. Many bloom-forming species, for instance *Prorocentrum* cf. *balticum*, *Ceratium* spp., and *Osteropsis* spp., produce vast amounts of muco-polysaccharide exudates, mucospheres, mucilage, or toxic mucus trap [168,169,170,171] (Figure 12A–C). Mucus production at these biomass levels could involve secretion of mucin-like proteins, as in the case of some diatoms [172], or potentially formed through partial hydrolysis after ecdysis; in either case, the amphiesma dynamics are involved. Whereas the AVs are circum-cortical with the flagella pores at longitudinal and transverse, the pusule could serve as special communication ports [173].

Mucocysts are single-membrane bound, flask-shaped vesicles that contain amorphous, finely granular material that are considered to form the mucosphere in the resting cyst. Trichocysts are rod-shaped extrusomal vesicles with proteinaceous twisted fibers (paracrystalline central body), which are responsible for the discharge of long trichocyst shafts that could potentially contribute to the mucus material [108,174]. Amorphous materials surrounded by the pusule membrane [175] could also be related to the mucus excretion observed for many species. The multiple “thecal pits and pores” in CTPs, which were speculated to be trichocyst openings, could have adopted such a role. pH was recognized to be a major factor in phytoplankton successions [176]. Bloom partial degradation with cellulosome(s) [177], or seawater CO_2_ uptake through microalgal respiration, would have increased local pH, likely altering the usual pH differentials, which will likely exert feedback on the population, especially in ecdysal propensity, in essence a passive quorum. The association of acidic compartments, as well as the endoglucanase dCel1p, with amphiesma will have implication to the ecdysed polysaccharides upon pellicle cyst formation.

## 11. Biomechanical Roles of Amphiesma

In addition to serving as barrier, or communication layer, with the extracellular environment, the amphiesmal-CTP/PL likely have multiple functional roles. Many dinoflagellates have relatively large cell sizes, and cortical CTP deposition represents substantial biomass. CTPs are an important component of marine snow, representing significant fixed carbon transport to the benthos [178]. The relative synchrony of CTP regeneration from ecdysal cysts (Figure 4A), represents an amendable single-cell system for the study of cellulose deposition without the primary–secondary cell wall asynchronicity and positional dependent development.

Nanoindentation studies suggested that CTP’s mechanical hardness levels were in the range of soft wood, with apparent homogeneity within the CTP board, but with higher strength at the CTP ridges [62]. Considering additional mechanical strength offered by the plate orthogonal assembly, as in the case of diatom valves [179], the thecal layer intuitively serves mechanical protection. The specific density of amorphous cellulose was estimated to be in the region of 1.4~1.5 g/cm^3^ [180], and the circum-cortical localization would render a potential ballasting-like function. Dinoflagellate swarmer cells whirl with movement of their longitudinal and transverse flagella (reviewed in [181]).

## 12. Conclusions

Amphiesmal dynamics thus orchestrate dinoflagellate cellular growth simultaneously with CTP/PL polysaccharide deposition, and its regulation dictates life-cycle transitions. It is apparent that the different bioprocesses, deflagellation–reflagellation, PL displacement–regeneration, and AV-CTP assembly, are modularly incorporated in their sequence of events during amphiesmal dynamics in different cell-life cycle transitions. The association of acidic compartments with _CTC_[Ca2+]^S^ suggests amphiesma is a major regulatory hub, having biochemical–biomechanical interactomes between the extracellular and intracellular environments. The significant roles of IP_3_-cADPR-Ca^2+^ signaling in cellular growth and amphiesmal dynamics, whether it is free-living or in symbiotic dinoflagellates, will be locally adapted in each cell type, with cortical chloroplasts representing a special axis. Amphiesma also serves as a communication hub between the external environment and the dinoflagellate cytosol, with many thecal pits and micropores speculated for nutrient exchanges. CTP formation encompasses carbon fixation, cellulose biogenesis, vesicular transport rate, quantitative Ca^2+^-membrane interactomes, optical biology, and spatial-temporal volume depletion force axis, in addition to dinoflagellate modelling in circadian rhythm, peridinin photobiology, and toxin biosynthesis; their developments with the ongoing genetic dissection and genome annotations will put amphiesma-CTP explorations at cross-forefronts between physical biology, biochemistry, synthetic biology, and molecular biology. 

## Data Availability

The data are available on request from the corresponding author.

## Supplementary Materials

The following supporting information can be downloaded at: https://www.mdpi.com/article/10.3390/md21020070/s1, Video S1: *Lingulodinium polyedrum* cell undergoing excystment, Video S2: Mucilage filament (sheath) produced by the benthic *Coolia* spp.
